# KSHV Seropositivity Associates With Impaired Immune Reconstitution in Virally Suppressed HIV

**DOI:** 10.1155/cjid/5156452

**Published:** 2026-08-23

**Authors:** Xiaowu Wang, Xiaoyu Fang, Tuantuan Li, Xinqi Geng, Yan Liu, Shuheng Wang, Dong Wu, Yilang Zhu, Linding Wang

**Affiliations:** ^1^ Department of Clinical Laboratory, The Second People’s Hospital of Fuyang City, Fuyang, Anhui, China; ^2^ Department of Clinical Laboratory, Fuyang Infection Disease Clinical College of Anhui Medical University, Fuyang, Anhui, China; ^3^ Department of Pharmacy, Jieshou City People’s Hospital, Fuyang, Anhui, China; ^4^ Anhui Provincial Key Laboratory of Zoonoses, Department of Microbiology and Parasitology, School of Basic Medical Sciences, Anhui Medical University, Hefei, Anhui, China, ahmu.edu.cn; ^5^ School of Mental Health and Psychology, Anhui Medical University, Hefei, Anhui, China, ahmu.edu.cn; ^6^ School of Public Health, Anhui Medical University, Hefei, Anhui, China, ahmu.edu.cn; ^7^ Department of Pharmacy, Fuyang People’s Hospital, Fuyang, Anhui, China

**Keywords:** combined diagnostic model, CD4^+^ lymphocyte count, HIV, immune reconstitution, IL-6, KSHV

## Abstract

**Background:**

Kaposi’s sarcoma–associated herpesvirus (KSHV) seropositivity is prevalent in people with HIV (PWH), yet its association with immune reconstitution during long‐term ART remains unclear.

**Methods:**

In this retrospective cohort study, we reviewed the records of 209 virally suppressed PWH (VL < 50 copies/mL) after ≥ 2 years of ART. Participants were stratified based on their KSHV serostatus (IgG+). Data on immune parameters were extracted from laboratory reports corresponding to the 2‐year post‐ART time point.

**Results:**

KSHV seropositive individuals exhibited significantly lower CD4^+^ counts (101.5 vs. 316.0 cells/μL; *p* < 0.001), reduced CD4/CD8 ratios (0.26 vs. 0.60; *p* < 0.001), and elevated IL‐6 (9.78 vs. 5.46 pg/mL; *p* < 0.001). Multivariate analysis identified IL‐6 as an independent predictor of seropositivity (OR = 1.238; *p* < 0.001). The combination of CD4^+^ (≤ 126 cells/μL) and IL‐6 (> 5.83 pg/mL) showed superior diagnostic accuracy for KSHV seropositivity (AUC = 0.832).

**Conclusion:**

KSHV seropositivity correlates with impaired immune reconstitution in virally suppressed PWH. CD4^+^ and IL‐6 may jointly serve as a screening tool for KSHV serostatus assessment, though their utility for detecting active infection requires further study.

## 1. Introduction

Kaposi’s sarcoma–associated herpesvirus (KSHV), the etiological agent of Kaposi sarcoma and lymphoproliferative disorders, exhibits high seroprevalence (15%–50%) in people with human immunodeficiency virus (HIV) (PWH) [1–3]. Although antiretroviral therapy (ART) effectively suppresses HIV replication and reduces viral load to undetectable levels, approximately 10%–40% of treated patients still fail to achieve adequate immune recovery clinically. These individuals are referred to as immunological nonresponders (INRs) [4]. Despite having achieved virological suppression, these patients exhibit persistently poor recovery of CD4^+^ T lymphocytes and incomplete immune reconstitution, hence being defined as INRs [5, 6]. Compared with immunological responders (IRs), INR patients have a significantly higher incidence of non–AIDS‐related events (such as malignancies and severe infections) and long‐term mortality, indicating a poorer clinical prognosis [5, 7]. Chronic herpesvirus coinfection has been proposed as a potential driver of persistent immune dysfunction in ART‐treated PWH [8, 9].

KSHV establishes lifelong latency with intermittent reactivation, potentially contributing to T‐cell exhaustion and systemic inflammation through viral interleukin‐6 (vIL‐6) homolog‐mediated pathways [10]. However, existing studies on KSHV and immune reconstitution primarily focus on untreated HIV or short‐term ART cohorts [11, 12]. The impact of KSHV serostatus—representing cumulative viral exposure—on long‐term CD4^+^ recovery and inflammatory milieu in virally suppressed PWH remains poorly characterized. This study aimed to (1) investigate the association between KSHV IgG seropositivity and immune reconstitution parameters (CD4^+^ count, CD4/CD8 ratio, and IL‐6) in PWH with ≥ 2 years of virologic suppression; (2) evaluate the diagnostic utility of combined CD4^+^ and IL‐6 profiling for identifying KSHV serostatus.

## 2. Materials and Methods

This retrospective cohort study was conducted at the Acquired Immune Deficiency Syndrome (AIDS) Specialty Department of the Second People’s Hospital of Fuyang City from June 1, 2023, to May 31, 2024. We enrolled 209 adult patients (≥ 18 years old) with confirmed HIV‐1 infection who met the following inclusion criteria: (1) receipt of continuous ART for ≥ 24 months with documented medication adherence (pharmacy refill rate > 95%); (2) sustained virologic suppression for ≥ 2 years, defined as two consecutive HIV‐1 RNA measurements < 50 copies/mL (with an interval of at least 6 months between measurements), and the most recent measurement was performed at the same visit as immune parameter assessment (24 months post‐ART initiation); HIV‐1 RNA was quantified using the Roche COBAS AmpliPrep/COBAS TaqMan HIV‐1 Test v2.0 (detection limit 20 copies/mL); and (3) complete demographic, clinical, and laboratory data records. Exclusion criteria included the following: (a) active comorbidities within 3 months prior to enrollment (e.g., *Pneumocystis jirovecii* pneumonia, cryptococcal meningitis, or active malignancies excluding Kaposi sarcoma); (b) confounding medical conditions (pregnancy, hepatitis B/C coinfection confirmed by HBsAg positivity or detectable HCV RNA, renal impairment with estimated glomerular filtration rate < 60 mL/min/1.73 m^2^, or hepatic dysfunction with alanine aminotransferase > 3 × upper limit of normal); and (c) incomplete immunological data at the 24‐month time point or discordant serological results. The study protocol was approved by the Institutional Ethics Committee of the Second People’s Hospital of Fuyang City. Written informed consent was waived in accordance with the Declaration of Helsinki due to the retrospective analysis of anonymized clinical data.

Laboratory methodologies were performed as follows. T lymphocyte subset analysis: Fresh EDTA‐anticoagulated peripheral blood samples (100 μL) were stained with a Mindray 4‐color monoclonal antibody cocktail (CD3‐fluorescein isothiocyanate/CD4‐phycoerythrin/CD8‐energy‐coupled dye/CD45‐phycoerythrin‐cyanin 5; specific catalog number) for 15 min at room temperature (20°C–25°C) protected from light. Erythrocytes were lysed using Mindray hemolysin solution (2.0 mL, 10‐min incubation). Analysis was performed on a Mindray BriCyte E6 flow cytometer (Shenzhen Mindray Bio‐Medical Electronics Co., Ltd., China) equipped with a 488‐nm excitation laser. Gating strategy: Lymphocyte population was identified by CD45 expression versus side scatter (SSC) properties, followed by quantification of CD3^+^/CD4^+^ and CD3^+^/CD8^+^ T‐cell subsets. Daily quality control included calibration with standard beads; the interassay coefficient of variation (CV) was < 6%.

IL‐6 quantification: Serum IL‐6 levels were measured using a quantum dot fluorescence immunoassay (QDFIA) on the KF‐Q002‐A Dry Fluorescence Immunoanalyzer (Shenzhen Jinzhun Biological Technology Co., Ltd., China). Briefly, 10 μL of serum was applied to test cartridges precoated with anti–IL‐6 capture antibodies. Quantum dot‐labeled detection antibodies (excitation/emission: 365/610 nm) generated fluorescence signals read at 15 min postincubation. Calibration was performed with manufacturer‐provided calibrators (Lot: KF‐Q002‐CAL). Assay sensitivity: 0.1 pg/mL; interassay CV: < 8%.

IL‐10 quantification: Serum IL‐10 levels were determined by enzyme‐linked immunosorbent assay (ELISA) using the Quantikine Human IL‐10 Immunoassay Kit (R&D Systems, Minneapolis, MN, USA; Catalog #D1000 B) according to the manufacturer’s protocol. Sensitivity: 1.5 pg/mL; inter‐assay CV: < 9%.

KSHV serostatus: IgG antibodies against KSHV were detected using a commercial ELISA kit (Human Herpesvirus‐8 IgG ELISA Kit, Wuhan Chundu Biological Technology Co., Ltd., China; Catalog #CD‐JK13166) according to the manufacturer’s instructions. This kit is intended for research use only. According to the manufacturer’s validation data (package insert), the assay has an intraassay CV of < 15% and an interassay CV of < 15%, with test validity requiring the average optical density (OD) of the positive control to be ≥ 0.8 and the negative control to be ≤ 0.2. Serum samples (50 μL/well) were incubated in antigen‐precoated microplates for 60 min at 37°C. After washing (5 cycles), horseradish peroxidase–conjugated antibodies were added. Color development used tetramethylbenzidine substrate, with OD measured at 450/620 nm. The cutoff value was calculated as the mean negative control OD + 0.25, and samples with OD ≥ cutoff were considered positive. All quality control criteria were met in each experimental run.

Statistical analysis was conducted using SPSS Statistics Version 26.0 (IBM Corp., Armonk, NY, USA) and MedCalc Statistical Software Version 20.0 (MedCalc Software Ltd., Ostend, Belgium). Continuous variables with non‐normal distribution (confirmed by the Shapiro–Wilk test, *p* < 0.05) were expressed as median and interquartile range (IQR) and compared using the Mann–Whitney *U* test. Categorical variables were presented as frequencies (%) and analyzed by Pearson’s *χ* ^2^ test or Fisher’s exact test as appropriate. To stabilize variance, CD4^+^ and CD8^+^ T‐cell counts were square‐root transformed prior to multivariate logistic regression analysis, as is standard practice in HIV immunology research. Multivariate binary logistic regression models were constructed to identify independent predictors of KSHV seropositivity, adjusting for age, sex, square‐root transformed CD4^+^ count, CD4/CD8 ratio, IL‐6 level, and significant comorbidities (hepatitis, tuberculosis, and cancer). Results were reported as adjusted odds ratios (ORs) with 95% confidence intervals (CIs). Diagnostic performance of biomarkers was evaluated by receiver operating characteristic (ROC) curve analysis, with optimal cutoff values determined by maximizing Youden’s index (sensitivity + specificity − 1). The combined diagnostic model was derived from logistic regression probability scores. Statistical significance was defined as two‐tailed *p* < 0.05. Post hoc power analysis indicated > 95% power to detect CD4^+^ count differences (effect size Cohen’s *d* = 0.7, *α* = 0.05).

## 3. Results

### 3.1. Baseline Characteristics of Study Subjects

A total of 209 participants were enrolled (161 HIV mono‐infected, 48 KSHV seropositive). No significant age difference was observed between KSHV‐seropositive and KSHV‐seronegative individuals (median 56.5 years [IQR 49.25–69.75] vs. 62.0 years [IQR 53.50–71.00], *p* = 0.091). KSHV seropositive subjects demonstrated significantly lower CD4^+^ T‐cell counts (median 101.5 cells/μL [IQR 48.25–272.75] vs. 316.0 cells/μL [IQR 170.50–484.50], *p* < 0.001) and reduced CD4/CD8 ratios (median 0.26 [IQR 0.08–0.47] vs. 0.60 [IQR 0.33–1.00], *p* < 0.001). IL‐6 levels were markedly elevated in seropositive subjects (median 9.78 pg/mL [IQR 6.62–15.14] vs. 5.46 pg/mL [IQR 4.87–5.82], *p* < 0.001). No significant differences were observed in other baseline characteristics (all *p* > 0.05) (Table 1). At the time of immune assessment, all 209 participants had undetectable HIV‐1 RNA (50 copies/mL), confirming sustained virologic suppression.

**TABLE 1:** Characteristics stratified by KSHV serostatus.

Characteristic	HIV (*n* = 161)	HIV/KSHV (*n* = 48)	*p* value
Age (years)	62.00 (53.50,71.00)	56.50 (49.25, 69.75)	0.091^a^
Gender, *n* (%)			
Male	59 (36.65)	15 (31.25)	0.493^b^
Female	102 (63.35)	33 (68.75)	
Geographical setting			
Yingzhou District	84 (52.17)	21 (43.75)	0.318^c^
Yingquan District	31 (22.98)	14 (29.17)	
Yingdong District	36 (22.36)	12 (25.00)	
Around Fuyang	4 (2.48)	1 (2.08)	
Occupation, *n* (%)			
Student	1 (0.62)	1 (2.08)	0.248^c^
Worker	17 (10.56)	8 (16.67)	
Farmer	128 (79.51)	34 (70.83)	
Retired	3 (1.86)	3 (6.25)	
None	12 (7.45)	2 (4.17)	
Hepatitis, *n* (%)	145 (90.06)	39 (81.25)	0.099^c^
Cancer, *n* (%)	151 (93.79)	47 (97.92)	
Syphilis, *n* (%)	150 (93.17)	42 (87.50)	
Tuberculosis, *n* (%)	143 (88.82)	44 (91.67)	
CD4, cells/μL, median (IQR)	316.00 (170.50, 484.50)	101.50 (48.25, 272.75)	< 0.001^a^
CD8, cells/μL, median (IQR)	500.00 (331.50, 713.50)	604.00 (346.50, 959.75)	0.138^a^
CD4/CD8 ratio, median (IQR)	0.60 (0.33, 1.00)	0.26 (0.08, 0.47)	< 0.001^a^
CD4, cells/μL			
> 500	37 (22.98)	4 (8.33)	0.001^c^
200–500	71 (44.10)	11 (22.92)	
100–200	31 (22.98)	9 (18.75)	
< 100	22 (13.66)	24 (50.00)	
CD4/CD8 ratio			
> 1	45 (27.95)	10 (20.83)	< 0.001^c^
0.5–1.0	56 (34.78)	9 (18.75)	
0.3–0.5	30 (18.63)	6 (12.50)	
< 0.3	30 (18.63)	23 (47.91)	
IL‐6, (pg/mL)	5.46 (4.87, 5.82)	9.78 (6.62, 15.14)	< 0.001^a^
IL‐10, (pg/mL)	4.87 (4.49, 6.57)	6.37 (4.66, 6.64)	0.153^a^

*Note:* KSHV, Kaposi’s sarcoma–associated herpesvirus; IL‐6, interleukin‐6; IL‐10, interleukin‐10; IQR, interquartile range. All *p* values are derived from univariate analyses and are not adjusted for multiple comparisons.

Abbreviation: HIV, human immunodeficiency virus.

^a^ *p* value obtained by the Mann–Whitney U test for continuous variables (all continuous data were nonnormally distributed as confirmed by the Shapiro–Wilk test, *p* < 0.05).

^b^ *p* value obtained by the Pearson’s χ^2^ test.

^c^ *p* value obtained by the Pearson’s χ^2^ test or Fisher’s exact test, as appropriate (Fisher’s exact test was used when expected cell counts were < 5).

### 3.2. Factors Associated With KSHV Seropositivity

Multivariate analysis identified IL‐6 as an independent predictor of KSHV seropositivity (OR = 1.238 per unit increase, 95% CI: 1.139–1.346; *p* < 0.001). CD4^+^ T‐cell count showed marginal significance (OR = 0.870, 95% CI: 0.810–0.980; *p* = 0.037), while the CD4/CD8 ratio was not independently associated (*p* = 0.161) (Table 2).

**TABLE 2:** Logistic regression analysis of factors associated with KSHV seropositivity.

Items	B	S.E.	Wald	*p* value	OR	OR 95% CI
√CD4^a^	−0.139	0.067	4.362	0.037	0.870	0.810–0.980
CD4/CD8	−1.025	0.731	1.968	0.161	0.359	0.086–1.502
IL‐6	0.214	0.043	25.12	< 0.001	1.238	1.139–1.346

*Note:* KSHV, Kaposi’s sarcoma–associated herpesvirus; IL‐6, interleukin‐6.

^a^CD4^+^ T‐cell counts were square‐root transformed prior to analysis to stabilize variance.

### 3.3. Diagnostic Performance of Biomarkers for KSHV Seropositivity

**FIGURE 1:**
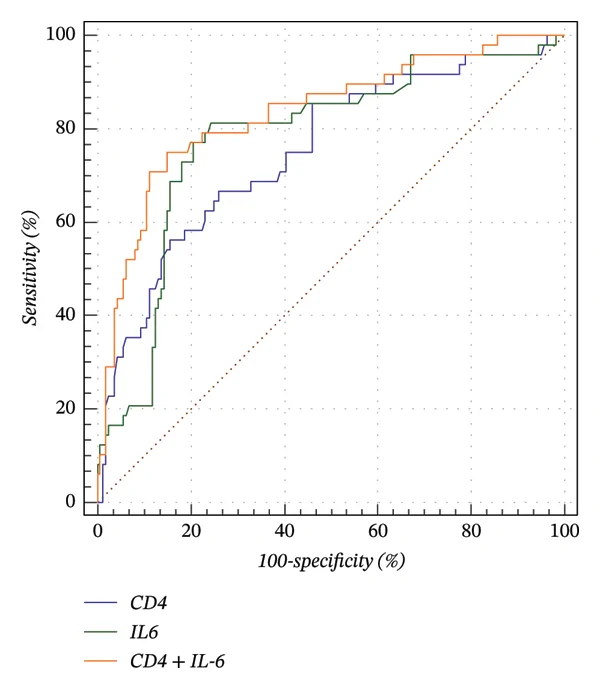
ROC curves for discriminating KSHV seropositive status. Active viral replication was not assessed.

ROC curve analysis revealed that IL‐6 (cutoff > 5.83 pg/mL, area under the curve [AUC] = 0.781) and CD4^+^ count (cutoff ≤ 126 cells/μL, AUC = 0.756) had significant value in identifying KSHV seropositivity (Figure 1). The combination of CD4^+^ count and IL‐6 improved diagnostic accuracy (AUC = 0.832, sensitivity 75.0%, and specificity 84.5%). This indicates that the combined model may serve as a screening tool for KSHV serostatus assessment (Table 3).

**TABLE 3:** Diagnostic efficacy of biomarkers for KSHV seropositivity.

Items	AUC	Cut off	Sensitivity (%)	Specificity (%)	95% *CI*	*p* value
CD4, (cells/μL)	0.756	≤ 126	68.75	73.29	0.692–0.812	< 0.001
IL‐6, (pg/mL)	0.781	> 5.83	81.25	75.78	0.719–0.835	< 0.001
CD4+IL‐6	0.832	_	75.00	84.47	0.765–0.873	< 0.001

*Note:* KSHV, Kaposi’s sarcoma–associated herpesvirus; IL‐6, interleukin‐6.

## 4. Discussion

In contrast to previous reports [11, 12], this study specifically enrolled individuals with sustained virological suppression for ≥ 2 years, thereby highlighting the enduring impact of KSHV on immune recovery even during ART‐mediated viral control.

The mechanisms underlying KSHV‐associated impairment of immune reconstitution are multifaceted. KSHV establishes lifelong latency with intermittent reactivation, expressing viral homologs of human cytokines such as vIL‐6. These factors may dysregulate host immune responses and promote chronic inflammation [10]. The significantly elevated IL‐6 levels observed in seropositive patients likely reflect both direct viral stimulation and downstream inflammatory cascades. Furthermore, KSHV may exacerbate T‐cell exhaustion and impair thymic output, contributing to the observed CD4^+^ lymphopenia and skewed CD4/CD8 ratios. Notably, although IL‐10—another cytokine modulated by KSHV—was slightly elevated in seropositive subjects, the difference lacked statistical significance. This observation suggests that IL‐6 may represent a more sensitive biomarker of KSHV‐associated immune dysregulation in this population.

The clinical relevance of KSHV coinfection is further underscored by emerging evidence linking it to systemic conditions beyond AIDS‐defining illnesses. For instance, a recent study from Xinjiang, China, reported a significant association between KSHV seropositivity and Type 2 diabetes mellitus [13]. Additionally, high seroprevalence rates have been documented in specific risk groups, such as men who have sex with men (MSM) with HIV in the southern United States, particularly associated with oral sexual practices and methamphetamine use [14–16]. These findings suggest that KSHV may exploit immunological, metabolic, and behavioral vulnerabilities to establish chronic infection and promote systemic inflammation.

Clinically, the combined use of CD4^+^ count (≤ 126 cells/μL) and IL‐6 level (5.83 pg/mL) achieved an AUC of 0.832 for discriminating KSHV seropositivity, indicating good diagnostic performance. This model represents a practical, low‐cost screening tool to identify KSHV seropositive individuals at elevated risk for incomplete immune reconstitution, who may consequently benefit from enhanced monitoring or more intensive management. However, it is crucial to note that seropositivity does not necessarily indicate active viral replication or clinical disease. Therefore, this model should be complemented by viral DNA testing when active KSHV infection is suspected.

It is important to distinguish between INRs, who exhibit impaired T‐cell function despite adequate CD4 counts, and individuals with poor CD4 reconstitution, as observed in our KSHV‐seropositive group. The latter reflects a quantitative deficiency in CD4^+^ T‐cell recovery despite sustained virologic suppression. In our cohort, the median CD4 count in KSHV‐seropositive individuals was only 101.5 cells/μL, indicating profound immunodeficiency despite undetectable HIV RNA at the time of assessment. This underscores the lasting impact of KSHV coinfection on CD4^+^ T‐cell homeostasis, even in the absence of active HIV replication.

Several limitations of this study warrant acknowledgment. First, the cross‐sectional design precludes causal inference regarding the relationship between KSHV serostatus and immune reconstitution. Second, the sample size, particularly within the KSHV seropositive group (*n* = 48), was relatively small, potentially limiting the generalizability of our findings. Third, due to limited residual serum volumes after performing the required serological and cytokine assays, we were unable to quantify KSHV viral load. Therefore, we could not distinguish between latent KSHV infection and active viral replication in seropositive individuals. Measuring KSHV‐specific T‐cell responses would also have provided deeper insights into host immunity dynamics. Future prospective studies with dedicated sample collection should include KSHV DNA testing to further elucidate the relationship between viral activity and immune reconstitution. Finally, although a commercially available ELISA kit was used for KSHV serology and the manufacturer reports acceptable performance characteristics (intraassay CV < 15%, interassay CV < 15%), we acknowledge that interassay variability and sensitivity limitations are well documented for KSHV serological tests in general [1]. Independent validation of this specific kit in a cohort of patients with active Kaposi sarcoma would further strengthen the assay’s performance assessment. Future studies should consider employing multiple serological algorithms or molecular methods to confirm KSHV infection status and reduce potential misclassification. Future studies should employ more robust serological algorithms or molecular methods to reduce misclassification.

In conclusion, this study provides compelling evidence that KSHV seropositivity is associated with significantly impaired immune reconstitution and heightened systemic inflammation in ART‐treated, virally suppressed PWH. The combined model utilizing CD4^+^ count and IL‐6 level offers a promising, clinically feasible approach for identifying KSHV seropositive individuals at elevated risk for poor immune recovery. These findings underscore the importance of considering KSHV coinfection in the management of PWH, particularly in regions with high KSHV prevalence, and lay the groundwork for future mechanistic and interventional studies.

## Author Contributions

Xiaowu Wang and Xiaoyu Fang contributed equally to this study. Xiaowu Wang designed the research framework, conducted the experimental procedures, and curated the data. Xiaoyu Fang performed the statistical analyses, interpreted the results, and assisted in manuscript preparation. Yan Liu, Xinqi Geng, and Shuheng Wang performed investigation. Yilang Zhu conceptualized the project, secured funding, supervised the entire investigation, and critically revised the manuscript for intellectual content.

## Funding

This work was supported by Fuyang City Health Committee scientific research project of 2023 (No. FY2023‐030).This study was supported by the Scientific Research Fund of Anhui Institute of Translational Medicine (2022zhyx‐C10) and Scientific Research Project of Higher Education Institutions in Anhui Province (2022AH040100).

## Disclosure

All authors reviewed and approved the final version of the manuscript.

## Conflicts of Interest

The authors declare no conflicts of interest.

## Data Availability

The data that support the findings of this study are available from the corresponding author upon reasonable request.

## References

[1] Nambiar P. , Liang T. , Labo N. et al., Kaposi Sarcoma-Associated Herpesvirus Risk and Disease in Kidney Donors and Transplant Recipients With HIV in the United States, Clinical Infectious Diseases. (2025) 5, no. 4, ciaf229–ciaf719, 10.1093/cid/ciaf229. PMC13110356 40324947

[2] Park S. Y. , Epling B. , Richey M. , Lupu D. , Mughar M. , and Sereti I. , Plasma Mcfdna Sequencing May Improve Usual Care Diagnostics to Detect KSHV Among Outpatient People With Advanced HIV, Pathog Immun. (2025) 10, no. 1, 187–197, 10.20411/pai.v10i1.788. 39959614 PMC11828093

[3] Ekenoğlu Merdan Y. , Merdan S. , and Etiz P. , Marked Temporal Decline in Human Herpesvirus-8 Seroprevalence Among People Living With HIV in Istanbul, Türkiye, Diagnostic Microbiology and Infectious Disease. (2025) 113, no. 4, 10.1016/j.diagmicrobio.2025.117037. 40753699

[4] Pang X. , He Q. , Huang J. et al., Characteristics and Influencing Factors of Immunological Non-Responders in HIV-1-Infected Patients Receiving Antiretroviral Therapy: A Cross-Sectional Study in Guangxi, Scientific Reports. (2024) 14, no. 1, 10.1038/s41598-024-79449-1. PMC11558013 39532976

[5] Yang X. , Su B. , Zhang X. , Liu Y. , Wu H. , and Zhang T. , Incomplete Immune Reconstitution in HIV/AIDS Patients on Antiretroviral Therapy: Challenges of Immunological Non-Responders, Journal of Leukocyte Biology. (2020) 107, no. 4, 597–612, 10.1002/JLB.4MR1019-189R. 31965635 PMC7187275

[6] Marchetti G. , Gazzola L. , Trabattoni D. et al., Skewed T-Cell Maturation and Function in HIV-Infected Patients Failing CD4+ Recovery Upon Long-Term Virologically Suppressive HAART, Acquired Immunodeficiency Syndrome. (2010) 24, no. 10, 1455–1460, 10.1097/QAD.0b013e328339cf40. 20539090

[7] Lapadula G. , Cozzi-Lepri A. , Marchetti G. et al., Risk of Clinical Progression Among Patients With Immunological Nonresponse Despite Virological Suppression After Combination Antiretroviral Treatment, Acquired Immunodeficiency Syndrome. (2013) 27, no. 5, 769–779, 10.1097/QAD.0b013e32835cb747. 23719349

[8] Deeks S. G. , Tracy R. , and Douek D. C. , Systemic Effects of Inflammation on Health During Chronic HIV Infection, Immunity. (2013) 39, no. 4, 633–645, 10.1016/j.immuni.2013.10.001. 24138880 PMC4012895

[9] Gianella S. , Massanella M. , Wertheim J. O. , and Smith D. M. , The Sordid Affair Between Human Herpesvirus and HIV, Journal of Infectious Diseases. (2015) 212, no. 6, 845–852, 10.1093/infdis/jiv148. 25748324 PMC4548466

[10] Ganem D. , KSHV Infection and the Pathogenesis of Kaposi’s sarcoma, Annual Review of Pathology: Mechanisms of Disease. (2006) 1, 273–296, 10.1146/annurev.pathol.1.110304.100133. 18039116

[11] Goncalves P. H. , Ziegelbauer J. , Uldrick T. S. , and Yarchoan R. , Kaposi Sarcoma Herpesvirus-Associated Cancers and Related Diseases, Current Opinion in HIV and AIDS. (2017) 12, no. 1, 47–56, 10.1097/COH.0000000000000330. 27662501 PMC6311702

[12] Bihl F. , Mosam A. , Henry L. N. et al., Kaposi’s Sarcoma-Associated Herpesvirus-Specific Immune Reconstitution and Antiviral Effect of Combined HAART/Chemotherapy in HIV Clade C-Infected Individuals With Kaposi’s Sarcoma, Acquired Immunodeficiency Syndrome. (2007) 21, no. 10, 1245–1252, 10.1097/QAD.0b013e328182df03. 17545700

[13] Cui M. , Fang Q. , Zheng J. et al., Kaposi’s Sarcoma-Associated Herpesvirus Seropositivity is Associated With Type 2 Diabetes Mellitus: A Case-Control Study in Xinjiang, China, International Journal of Infectious Diseases. (2019) 80, 73–79, 10.1016/j.ijid.2019.01.003. 30639407

[14] Tsai M. J. , Sun H. Y. , Hsieh S. M. et al., Seroepidemiology of the Human Herpesvirus 8 Infection Among People Living With HIV in Taiwan, 2014–2018, Journal of Microbiology, Immunology, and Infection. (2021) 54, no. 5, 934–943, 10.1016/j.jmii.2020.11.005. 33349600

[15] Knights S. M. , Salyards M. , Kendall N. et al., High Seroprevalence of Kaposi Sarcoma-Associated Herpesvirus in Men Who Have Sex With Men With HIV in the Southern United States, Open Forum Infectious Diseases. (2023) 10, no. 4, 10.1093/ofid/ofad160. PMC10122490 37096147

[16] Liu Z. , Fang Q. , Zuo J. et al., Global Epidemiology of Human Herpesvirus 8 in Men Who Have Sex With Men: A Systematic Review and Meta-Analysis, Journal of Medical Virology. (2018) 90, no. 3, 582–591, 10.1002/jmv.24960. 28975631

